# Screening for chitin degrading bacteria in the environment of Saudi Arabia and characterization of the most potent chitinase from *Streptomyces variabilis* Am1

**DOI:** 10.1038/s41598-023-38876-2

**Published:** 2023-07-20

**Authors:** Essam Kotb, Amira H. Alabdalall, Azzah I. Alghamdi, Ibtisam M. Ababutain, Sumayh A. Aldakeel, Safa K. Al-Zuwaid, Batool M. Algarudi, Sakina M. Algarudi, Asmaa A. Ahmed, Ahmed M. Albarrag

**Affiliations:** 1 Basic and Applied Scientific Research Center (BASRC), Imam Abdulrahman Bin Faisal University (IAU), P.O. Box 1982, Dammam, 31441 Saudi Arabia; 2 Department of Biology, College of Science, Imam Abdulrahman Bin Faisal University (IAU), P.O. Box 1982, Dammam, 31441 Saudi Arabia; 3 The National Center for Genomic Technology (NCGT), Life Science and Environment Research Institute, King Abdulaziz City for Science and Technology (KACST), Riyadh, Saudi Arabia; 4 Genomic of Infectious Diseases Laboratory, Saudi Center for Disease Prevention and Control, Public Health Authority, Riyadh, Saudi Arabia; 5 Department of Statistics, Faculty of Commerce, Al-Azhar University (Girls’ Branch), P.O. Box 11751, Cairo, Egypt; 6 Department of Pathology, School of Medicine, King Saud University, Riyadh, Saudi Arabia

**Keywords:** Applied microbiology, Enzymes, Isolation, separation and purification, Microbiology techniques

## Abstract

Forty-six promising chitinolytic isolates were recovered during a screening for chitinolytic bacteria in the environment of Saudi Arabia. The top three isolates belonged to the genus *Streptomyces*. *Streptomyces variabilis* Am1 was able to excrete the highest amount of chitinases, reaching the maximum at 84 h with 0.5% yeast extract and nitrogen source and 2% galactose as a carbon source. Purification of chitinase by DEAE-Cellulose and Sephadex G_75_ improved the specific activity to 18.6-fold and the recovery to 23.8% and showed a mass at 56 kDa. The optimal catalysis of the purified chitinase was at 40 °C and pH 8 with high thermostability and pH stability as reflected by a midpoint temperature value of 66.6 °C and stability at pH 4–9. The protein reagents SDS, EDTA, and EGTA significantly inhibited the enzyme and the EDTA-chelated chitinase restored its activity after the addition of Fe^2+^ ions suggesting a metallo-chitinase type with ferric ions as cofactors. Chitinase exerted high antifungal activity against some phytopathogenic fungi. Interestingly, the tested *Streptomyces* were able to produce chitosan nanocubes along with chitosan from chitin degradation which may be an additional power in their antifungal activity in nature. This work also reveals the importance of unexplored environments as a pool of promising microorganisms with biotechnological applications.

## Introduction

One of the richest biopolymers found in the world is chitin. It consists of N-acetyl D-glucosamines (NAG) connected by β-1,4-glycosidic linkages. It supports the outer protective layer of many organisms including insects, fungi, and other crustaceans^1^. Additionally, eggshells of nematodes such as *Globodera rostochiensis* and *Meloidogyne javanica* are composed of 9 and 30% of chitin, respectively^2^. Chitin protects these pathogenic, parasitic, and disease-causing organisms, from extreme environmental conditions and the host's defensive mechanisms, therefore, facilitating parasitism and the spread of these organisms to other hosts and other localities. Regarding phytopathogenic fungi, crop losses could range up to 5–25% in developed countries whereas in undeveloped countries crop losses may reach 20–50%^3^.

Chitinases are those enzymes breaking down chitin by hydrolyzing β-1,4-glycosidic bonds converting it to chitooligosaccharide (COS), further action of chitobiases leading to NAG. Depending on their activity, chitinases can be categorized into exochitinases and endochitinases subgroups^4^. In general, chitinases have a molecular mass of 20–120 kDa^5^. They are within the glycoside hydrolase groups (GH) GH18 and GH19. Mammals, fungi, and bacteria are observed to accommodate chitinases in the GH18 family. Whereas some bacteria and higher plants contain chitinases in the GH19 family^6^.

Interestingly, chitinases are produced by all arthropods including pathogenic and non-pathogenic species to play a significant role in the ecdysis or molting process of the current cuticle and helps in the production of newer one. Additionally, they are present in the salivary glands of several insects to be used in the breakdown of the host cuticle^7^. Also, several fungi can produce chitinases to be utilized for their nutrition, morphogenesis, development, and as a defense mechanism against other organisms containing chitin. These species include *Saccharomyces cerevisiae* and some filamentous fungi such as *Trichoderma* sp., *Penicillium* sp., *Lecanicillium* sp., *Aspergillus* sp., *Stachybotrys* sp. and *Agaricus* sp. Moreover, some nematodes such as *Caenorhabditis elegans* produce chitinase as a form of defense against surrounding competitive species^2^.

Chemical insecticides, fungicides, and nematicides are the main tools for managing these pathogens. However, because of their detrimental impact on animals and humans, and the environment, novel techniques are being introduced. One of the most important strategies is to target these pathogens with chitinases produced by bacteria such as *Bacillus*, *Pseudomonas, Enterobacter, Serratia, and Streptomyces*^8^.

Genus *Streptomyces* is a common producer of several hydrolytic enzymes including chitinases. Chitinases were successfully produced from *Streptomyces venezuelae*^9^, *Streptomyces halstedii*^10^, *Streptomyces viridificans*^11^, *Streptomyces aureofaciens*^12^, *Streptomyces* sp. ANU6277^13^, *Streptomyces* sp. M-20^14^, *Streptomyces* sp. DA11^15^, and *Streptomyces* sp. S-84^16^. Concerning *Streptomyces mutabilis*, only two strains were preliminarily checked before for chitinase productivity along with other metabolites while studying several actinobacterial isolates from the Algerian Sahara^17^ and endophytic actinobacteria from the native plant roots in France^18^. Furthermore, chitinase enzyme hasn’t been fully characterized in *Streptomyces mutabilis* once before.

The production of chitinases from these microorganisms is not only employed in the biological control of pests but also utilized for various purposes including biomedical, industrial, and environmental applications. Additionally, providing aid in cosmetics, and flavor enhancement. For example, chitinous wastes from the food industry such as shrimp and crab shells are degraded using chitinases to accomplish high-quality food and feed products such as COS^19^.

Chitinases as a biocontrol agent against phytopathogenic fungi, successfully inhibited many disease-causing fungi such as *Pestalotia theae*, *Rhizoctonia solani*, *Botrytis cinerea,* and *Bipolaris oryzae*, thereby displaying antimicrobial activity^20,21^. The current study covers the green production of chitinase from bacterial sources such as *Streptomyces variabilis* as a potential tool against crop-threatening phytopathogenic fungi. According to our information, this is the first research on enzyme characterization of *Streptomyces variabilis* chitinase.

## Results

### Screening of isolates for their chitinolytic activity

During a screening for chitinolytic microorganisms in the terrestrial environment of Saudi Arabia, we obtained more than forty-six promising isolates. The chitinolytic activity on chitin agar was confirmed in 21.2% of isolates. The highest number of chitinolytic isolates (15%) was recorded for samples collected from the rhizosphere region of natural plants growing in the Nafud desert, Saudi Arabia. The top three isolates Nof21, Am1, and Bat2 were recovered from the root region of wild plants growing in the Great Nafud desert, Dahna desert, and North Shaqra desert of Saudi Arabia, respectively. Collection of soil samples were done from September to December 2021.

**Figure 1:**
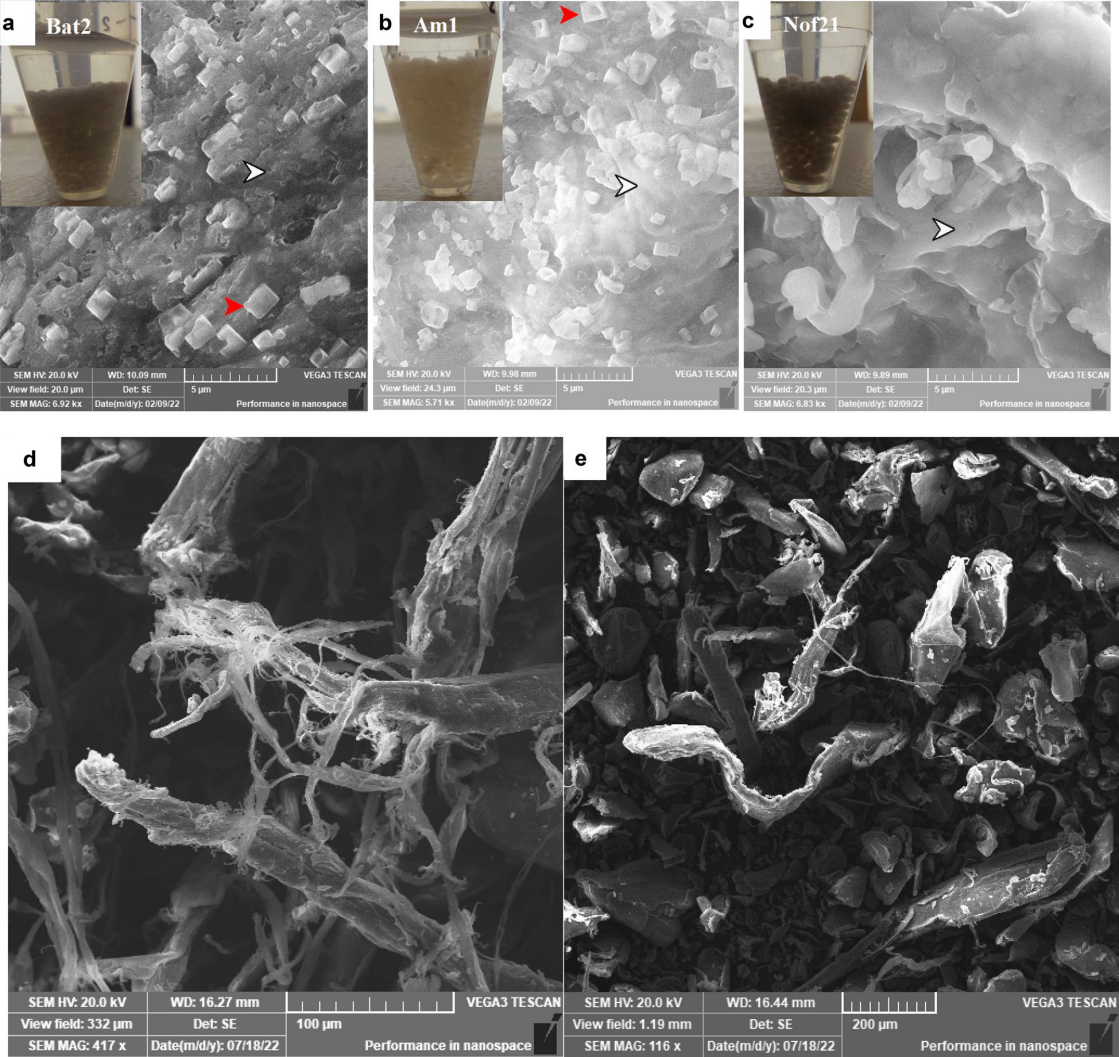
Characteristic beads observed inside the fermentation broth after 6 days of fermentation (upper left of panel **a**, **b**, and **c**). Examination of beads under SEM declared the formation of chitosan (white arrowhead) and chitosan nanocubes (red arrowhead) especially in case of strain Bat2 and strain Am1. Panel (**d**) represents the chitin loosening and degradation during the course of chitin degradation by isolate Am1 while, Panel (**e**) represents chitin before the enzymatic hydrolysis.

The tested species of *Streptomyces* were able to produce chitosan and chitosan nanocubes (NCs) from chitin degradation (Fig. 1a–e). Interestingly, during the assessment of enzymatic productivity among the positive isolates, characteristic beads (Fig. 1 upper left) appeared along with the fermentation broth. These beads were examined under SEM which revealed the formation of chitosan and the formation of chitosan nanocrystals with chitosan in the case of Bat2 and Am1 (Fig. 1c). The chitosan NCs of isolate Am1 were characterized by prominent invaginations at their centers. In terms of chitinase productivity, isolate designated as Nof21 was selected for the completion of the current study.

**Figure 2:**
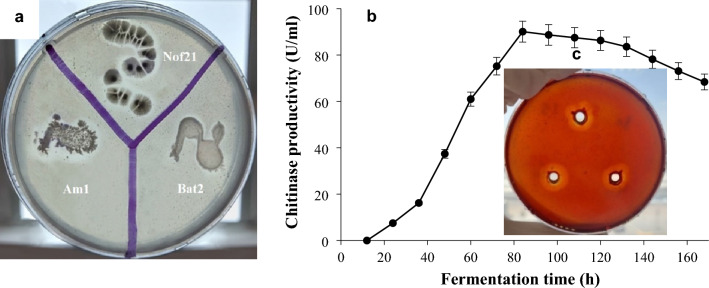
(**a**) Chitinolytic activity of some local isolates belonging to actinomycetes on chitin agar composed of colloidal chitin (10.0 g/l), NH_4_Cl (5 g/l), MgSO_4_.7H_2_O (0.5 g/l), KH_2_PO_4_ (2.4 g/l), K_2_HPO_4_ (0.6 g/l), and bacteriological agar (15 g/l). The medium was adjusted to pH 7.0 and incubation of cultures was done at 30 °C up to 6 days. Clear zones are indicative of chitinase production and hydrolysis of chitin. (**b**) Time course of enzyme production. (**c**) assay of chitinase in chitin agar petridish containing agar (1.5%, w/v) and colloidal chitin (1%, w/v) at pH 7.0 (**b**). Forty microliters of chitinase were applied to each well (7 mm diameter). By the end of incubation period a thin layer of Lugol’s iodine solution was applied on the surface of petridish for 10 min for visualization of the enzymatic reaction. Simple sugars resulted from chitin degradation don’t absorb the iodine solution while undegraded chitin absorb the dye and appears red.

Regarding chitinase production, the most potent isolates (Fig. 2a) were characterized molecularly based on *16SrDNA* gene fingerprint as *Streptomyces mutabilis* Nof21 (accession number: OP647097), *Streptomyces variabilis* Am1 (accession number: OP647095), and *Streptomyces roietensis* Bat2 (accession number: OP647096).

### Chitinase production

The enzyme was produced using the basal medium described in materials and methods at pH 6 and 35 °C for up to 6 days. Figure 2b displays the impact of time on enzyme production by *Streptomyces variabilis* Am1. Enzyme production enhanced with time during the logarithmic phase of bacterial growth achieving the highest value after 84 h (110.2 U/ml)*.* While the best bacterial growth was reached on the 5th day of incubation (24 mg dry weight/1 ml broth).

**Figure 3:**
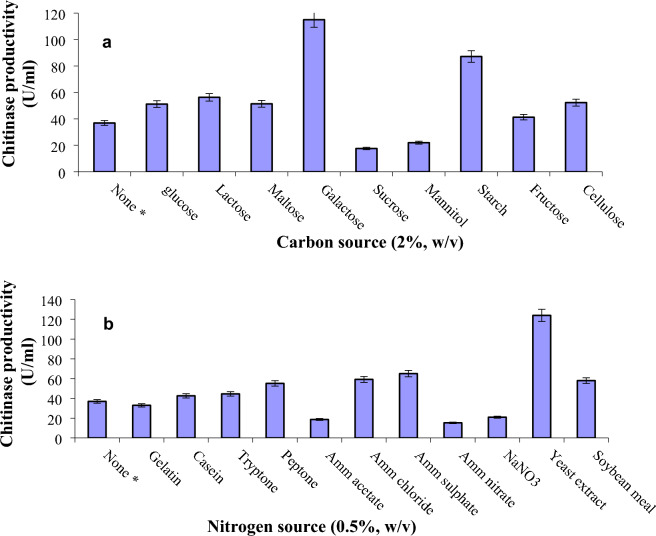
Effect of carbon source (**a**) and nitrogen source (**b**) on chitinase production. The initial fermentation medium was consisting of colloidal chitin (10 g/l), (NH_4_)_2_SO_4_ (5 g/l), MgSO_4_.7H_2_O (0.5 g/l), KH_2_PO_4_ (2.4 g/l), K_2_HPO_4_ (0.6 g/l), NaCl (0.6 g/l), FeSO_4_.7H_2_O (0.001 g/l), ZnSO4. 7H_2_O (0.0001 g/l), and MnSO4. 7H_2_O (0.0001 g/l). During the nitrogen source experiment, (NH_4_)_2_SO_4_ was replaced with the tested material. The basal medium was adjusted to pH 6.0 and the fermentation was done at 35 °C for 6 days in a submerged mode with 150 rpm shaking speed.

The impact of carbon sources such as cellulose, lactose, glucose, galactose, fructose, maltose, mannitol, sucrose, and soluble starch was examined at a concentration of 2% (w/v). Figure 3a shows that, best chitinase productivity by *Streptomyces variabilis* Am1 was achieved by galactose where enzyme productivity was 3.1-fold greater than the blank test. On the other hand, both sucrose and mannitol have repressed the chitinase productivity compared with the control treatment.

The influence of organic nitrogen such as casein, gelatin, soybean meal, tryptone, yeast extract, and peptone in addition to the inorganics such as ammonium acetate, ammonium nitrate, ammonium sulfate, ammonium chloride, and NaNO_3_, on chitinase secretion, were tested at 0.5% (w/v) concentration (Fig. 3b). Maximum chitinase productivity was achieved by yeast extract with a 3.3-fold increase over that of control. None of the ammonium nitrate, ammonium acetate, and sodium nitrate could enhance chitinase productivity by the tested strain (Fig. 3b).

### Properties of the purified chitinase

**Figure 4:**
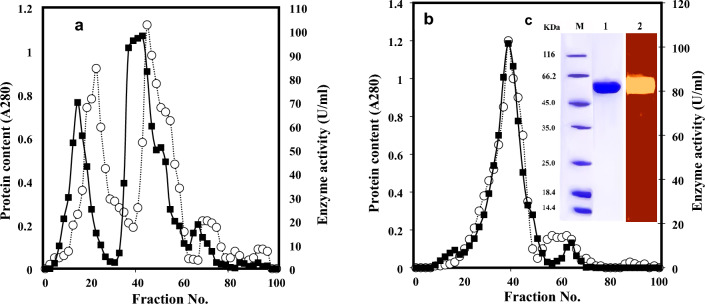
Chromatogram of the chitinase produced by *Streptomyces variabilis* Am1 on a DEAE-Cellulose column (2 × 30 cm^2^) (**a**). The pooled active fractions from the DEAE-Cellulose column were further purified with a Sephadex G_75_ FF column (2.0 × 45 cm^2^) (**b**). The -■- line represents chitinase activity, while -○- line represents the amount of protein in terms of absorbance at wavelength 280 nm. The last recovered protein was subjected to SDS-PAGE (**c**) with 5% (w/v) stacking gel and 15% (w/v) resolving gel. M represents the standard proteins, lane 1 represents the purified enzyme on SDS-PAGE gel, while lane 2, represents the activity gel electrophoresis (zymogram). The native gel containing contained 1.0% colloidal chitin as substrate, the gel was immersed in 0.2 M Tris–HCl buffer, pH 8.0 at 40 °C for 16 h. Chitinase activity in the native gel was visualized by staining with Lugol’s iodine solution for 15 min and destaining with 1 N NaCl solution for 5 min at room temperature. Full-length gel is included in the Supplementary Information file (Fig. S1).

Optimization of enzyme productivity by the most potent isolate *Streptomyces variabilis* Am1 was done to proceed with enzyme purification. The chitinase was refined to homogeneity by anion exchange chromatography (Fig. 4a) and gel permeation chromatography (Fig. 4b), respectively after being precipitated by 80% ammonium sulfate. The recovery of enzyme reached 23.8% and the specific activity reached 18.6-fold (Table 1). SDS-PAGE presented one band at a molecular mass of approximately 56 kDa (Fig. 4c Lane 1). Zymogram analysis (Fig. 4c Lane 2) of the culture supernatant also showed one band of enzymatic activity.

**Table 1:** Summary of enzyme purification steps.

Purification step	Total activity (U)	Specific activity (U/mg)	Recovery (%)	Purification (fold)
Culture broth	4125.1	13.2	100.0	1.00
(NH_4_)_2_SO_4_ precipitate	3546.3	31.8	86.0	2.4
Anion exchange (DEAE-Cellulose)	1423.2	78.1	34.5	5.9
Gel filtration (Sephadex G_75_ FF)	980.6	245.7	23.8	18.6

The amount of proteins was quantified (mg/ml) by subtracting the value 0.76 *A*_260_ from 1.55 *A*_280_. The enzyme recovery was calculated by dividing the total enzyme activity by the starting total activity, then multiplying by 100. The purification fold (*x*) was calculated by dividing the specific activity by the starting value.

**Figure 5:**
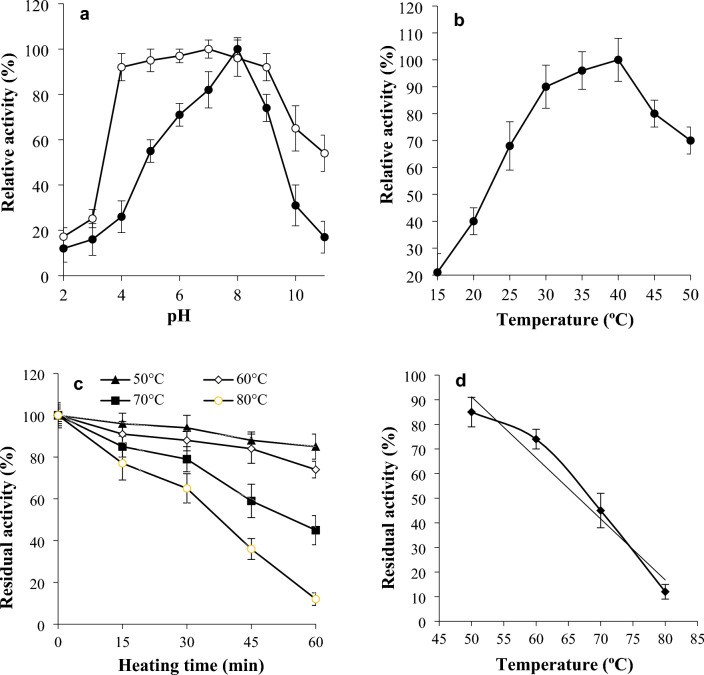
Effect of pH on enzyme activity (-●-) was done by adjusting the reacting mixtures to different pHs (**a**). While the pH stability (-○-) was examined by preincubation of enzyme at different pHs, then residual activity against colloidal chitin was determined (**a**). The effect of temperature on chitinase activity (**b**) was studied by incubation of the reacting mixtures at different temperatures. While the thermal stability (**c**) was determined by exposing chitinase to 50–80 °C for 15–60 min then assayed for residual activity against colloidal chitin. The midpoint temperature (*T*_m_) at which the tested chitinase lost half of its activity was calculated from (**d**).

The optimum pH for chitinase was 8.0 and chitinase was stable at pH 4–9 for 60 min at 35 °C (Fig. 5a). The best temperature for chitinase was 40 °C (Fig. 5b) and the enzyme was stable for 30 min under 60 °C (Fig. 5c). It appeared that chitinase wasn’t altered by preincubating at 50 °C for 15 min. From the thermal inactivation profile (Fig. 5c), the *T*_*m*_ value of chitinase was at 66.61 °C using 50 mM phosphate buffer (pH 8.0) (Fig. 5d).

### Impact of metals and protein reagents on chitinase activity

Results in Table 2 show the effect of metal ions such as Mg^2+^*,* Cu^2+^*,* Ca^2+^, Mn^2+^*,* Hg^2+^*,* Ag^+^*,* Fe^2+^*,* and Fe^3+^ in addition to β-mercaptoethanol*,* SDS, EGTA*,* and EDTA on chitinase activity at 5 mM concentration. In all treatments, a universal blank was made for comparisons. The enzyme was slightly inhibited by Cu^2+^ and Mn^2+^ ions while was stimulated by Fe^2+^ ions. The protein reagents SDS, EDTA, and EGTA significantly inhibited the tested chitinase. Interestingly the EDTA-chelated enzyme restored its activity upon the addition of Fe^2+^ ions at 5 mM concentration.

**Table 2:** Effect of metal ions and chemical reagents on chitinase activity.

Material	Relative activity (%)	Material	Relative activity (%)
None	100 ± 0.0	Fe^2+^	123 ± 3
Mg^2+^	103 ± 7	Fe^3+^	102 ± 5
Cu^2+^	72 ± 5	β-mercaptoethanol	65 ± 2
Ca^2+^	96 ± 6	SDS	34 ± 1
Mn^2+^	74 ± 4	EGTA	17 ± 1
Hg^2+^	89 ± 3	EDTA	3 ± 1
Ag^+^	101 ± 5	EDTA + Fe^2+^	92 ± 6

### Antifungal activity

**Figure 6:**
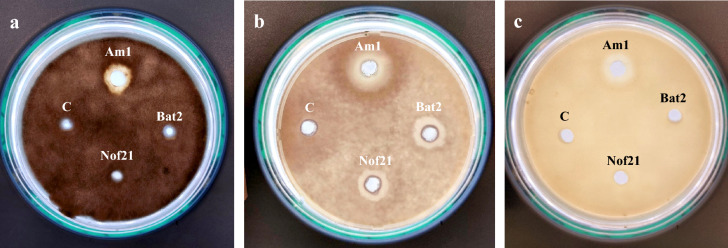
The antifungal activity of three chitinases produced by local *Streptomyces* spp (Am1, Bat2 and Nof21) against *Eurotium amstelodami* (**a**), *Fusarium verticillioides* (**b**), and *Fusarium oxysporum* (**c**). Exactly 40 µl (15 U) of enzyme preparations were applied to each well. A heat-inactive enzyme preparation was used as a negative control. Fungal growth was observed periodically till 7 days of incubation at 30 °C.

After 7 days of incubation of *Eurotium amstelodami*, *Fusarium oxysporum, and Fusarium verticillioides* on PDA plates with each well loaded with 40 µl of the enzyme preparation containing 15 U of chitinase, the antifungal potential of tested chitinases was calculated in millimeters (mm) utilizing a digital caliber and calculating the zones devoid of fungal growth around the hole. The best antifungal activity (Fig. 6) was reported for chitinase produced from *Streptomyces variabilis* Am1 where it exerted good inhibitory results against all tested fungi; *Eurotium amstelodami* (14.28 mm), *Fusarium verticillioides* (22.40 mm), and *Fusarium oxysporum* (16.18 mm). The chitinases of *Streptomyces mutabilis* Nof21 and *Streptomyces roietensis* Bat2 only inhibited the plant pathogenic fungus *Fusarium verticillioides* (14.20 and 14.98 mm, respectively)*.* The heat-inactivated chitinase didn’t show activity against any of the tested phytopathogenic fungi.

## Discussion

Chitinases are very useful in the degradation of chitin wastes and in the biological control of chitin containing pathogens such as fungi, insects and nematodes. Hence, screening for chitinolytic bacteria was performed in the terrestrial environments of Saudi Arabia. We obtained more than forty-six promising isolates. The most potent isolates were characterized based on *16SrDNA* gene fingerprint as *Streptomyces mutabilis*, *Streptomyces variabilis*, and *Streptomyces roietensis*. Regarding previous studies, very few authors discussed the ability of these species to produce chitinases. The ability of *Streptomyces variabilis* to produce chitinase was only detected in two strains; strain LCP18^22^ and strain YH21^23^. To our knowledge, chitinase production from *Streptomyces mutabilis* and *Streptomyces roietensis* has never been reported.

In terms of enzyme productivity, *Streptomyces variabilis* was selected for completion of this study. Enzyme production enhanced with time during the logarithmic phase of bacterial growth achieving the highest value after 84 h (110.2 U/ml)*.* While the best bacterial growth was reached on the 5th day of incubation (24 mg dry weight/1 ml broth). Fermentation was best by *Streptomyces venezuelae* P10 at 30 °C after 96 h^9^ while, *Streptomyces* sp. ANU6277 produced the maximum level of chitinase at 35 °C after 60 h of fermentation^13^.

The best productivity by *Streptomyces variabilis* Am1 was achieved in presence of galactose as a carbon source (3.1-fold greater than the blank test). According to other investigators, in the case of *Streptomyces halstedii*, chitinase production was highly potent in the presence of glucose^10^, arabinose in the case of *Streptomyces viridificans*^11^, and starch in the case of *Streptomyces* sp. ANU6277^13^ and *Streptomyces aureofaciens*^12^.

Maximum chitinase productivity was achieved by yeast extract with a 3.3-fold increase over that of control. None of the ammonium nitrate, ammonium acetate, and sodium nitrate could enhance chitinase productivity by the tested strain. Yeast extract was also the optimal N-supply for chitinase productivity from *Streptomyces* sp. ANU6277^13^.

When chitinase was refined to homogeneity by anion exchange chromatography and gel permeation chromatography, it showed a molecular mass of approximately 56 kDa by SDS-PAGE (Fig. 4c, lane 1) and activity zymographic gel electrophoresis (Fig. 4c, lane 2). In general, chitinases produced by microorganisms have a molecular mass ranging from 20 to 120 kDa. Fungal chitinases have a weight ranging from 35 to 45 kDa^24^ while most bacterial chitinases mostly weight around 20–60 kDa^25^. Chitinases of *Streptomyces* weight 20 kDa for *Streptomyces* sp. M-20 (Kim et al. 2003)^14^, 28 kDa, 35 kDa, and 45 kDa for *Streptomyces* sp. NK 1057 (Nawani and Kapadnis 2004)^26^, 34 kDa for *Streptomyces* sp. DA11 (Han et al. 2009)^15^, 43 kDa and 45 kDa for *S. albovinaceus* S-22^27^, 45 kDa for *Streptomyces* sp. ANU6277^13^ and 49 kDa for *S. griseus* HUT 6037^28^ and 66 kDa for *Streptomyces venezuelae* P10^9^.

The optimum pH for chitinase was 8.0 and chitinase was stable at pH 4–9 for 60 min at 35 °C (Fig. 5a). The best temperature for chitinase was 40 °C (Fig. 5b) and the enzyme was stable for 30 min under 60 °C (Fig. 5c). Previously, it was found the optimum reaction temperature for chitinases from *Streptomyces* specifically at 40 °C^29^ and in the range of 30–55 °C in general^30^. *Streptomyces* sp. M-20 chitinase exerted its maximal activity at 30 °C and pH 5.0 in addition, it was stable at pH 4.0–8.0 and thermally stable up to 40 °C^14^. *Streptomyces venezuelae* P10 chitinase was maximally active at 35 °C and pH 6–8^9^. While the chitinase of *Streptomyces* sp. DA11 was maximally active at 50 °C and pH 8^15^.

We suggest a metallo-enzyme type with ferric ions as cofactors because the EDTA-chelated chitinase restored its activity upon the addition of Fe^2+^ ions. We don’t suggest chitinase with thiol groups as there was no increase in activity upon treatment with β-mercaptoethanol and Hg^2+^ ions. The chitinase inhibition by SDS was also reported by Groleau et al.^31^. The inhibition of enzyme by chelating agents EGTA and EDTA was also reported by *Streptomyces* sp. DA11 chitinase^15^ and was different with *Streptomyces* sp. M-20 chitinase^14^, and *Streptomyces* sp. S-84 chitinase^16^.

*Streptomyces variabilis* Am1 chitinase exerted good inhibitory results against all tested fungi; *Eurotium amstelodami*, *Fusarium verticillioides*, and *Fusarium oxysporum*. The dependence on the hydrolytic activity of microbial chitinolytic enzymes in the biocontrol of harmful fungi especially the phytopathogenic type is well-established in many studies. Chitinolytic enzymes from actinobacteria especially genus *Streptomyces* were found to have various mechanisms of antifungal activity such as inhibition of fungal spore germination, inhibition of germ tube elongation, and induction of spores and hyphal tips bursting^12,32^. The application of bacterial chitinases in antifungal applications was first reported from *Streptomyces griseus* HUT 6037^33^. *Streptomyces venezuelae* P10 chitinase was found to inhibit *Aspergillus niger* maximally but didn’t inhibit *Penicillium chrysogenum*^9^. *Streptomyces* sp. DA11 chitinase was employed for the biocontrol of *Aspergillus niger* (11.0 mm) and *Candida albicans* (10.5 mm)^15^. Hoster et al.^29^ found chitinase activity from a strain of *Streptomyces* against *Fusarium culmorum*, *Aspergillus nidulans*, *Gulgnardia bidwellii*, *Botrytis cinerea*, and *Sclerotia sclerotiorum*. Narayana and Vijayalakshmi^13^ reported the chitinolytic activity of *Streptomyces* sp. ANU6277 against *Fusarium udum*. Kim et al.^14^ revealed the inhibition of the phytopathogen *Botrytis cinerea* by chitinase from *Streptomyces* sp. M-20*.*

The ability of tested species of *Streptomyces* to produce chitosan and chitosan NCs from chitin degradation (Fig. 1a–e) may be additional tools in their antifungal activity besides the hydrolysis activity of chitinase. Regarding other investigators, Chitosan was used by many researchers as an antibacterial and antifungal agent due to its polycationic nature that facilitates adsorption to the negatively charged cellular components on the surface of pathogenic microorganisms^34^.

Further degradation of chitosan into chitosan nanoparticles (NPs) and NCs enhances its antifungal potential owing to improved surface area and encapsulation proficiency^35^. In addition, exposing microbes including fungi to metal nanoparticles creates oxidative stress in the form of reactive oxygen species that disrupts cell selective permeability, and inhibits nucleic acids, and protein synthesis.

Chitosan NPs and NCs were prepared by chemical and physical methods and used by agro-researchers as antifungals to substitute the use of chemical fungicides. For example, Chouhan et al.^36^ have controlled the *Fusarium* rot of wheat by chemically prepared chitosan NPs. Biologically prepared NPs and NCs are eco-friendly, biocompatible, have superior permeability into the cell, lower toxicity, and lower cost^37^. Regarding other researchers, a conjugate of chitosan NPs and silver NPs was used as an antifungal composite against *Fusarium* species. Unfortunately, it was expensive and became unsuitable for field application in agriculture^38^. The value of this research is that it is the first time to produce chitosan NPs and NCs utilizing Streptomyces. The nanocrystals in particular have superior activity than other forms of nanoparticles.

As a conclusion, forty-six chitinolytic isolates were recovered from the rhizosphere of some desert plants in Saudi Arabia. The top three were recovered from the root region of wild plants growing in the Great Nafud desert, Dahna desert, and North Shaqra desert and then were identified as *Streptomyces mutabilis* Nof21, *Streptomyces variabilis* Am1, and *Streptomyces roietensis* Bat2, respectively. According to our knowledge, chitinases production from *Streptomyces mutabilis* and *Streptomyces roietensis* have never been reported, in addition, chitinase characterization from *Streptomyces variabilis* has never been done. Interestingly, SEM revealed the formation of chitosan NCs along with chitosan from the degradation of chitin. The ability of tested species of *Streptomyces* to produce chitosan and chitosan NCs from chitin degradation may be additional tools in their antifungal activity besides the hydrolysis activity of chitinase. Chitosan itself is an antimicrobial agent due to its ability to adsorb the cells of pathogenic microorganisms. Chitosan NCs enhance the antifungal potential owing to improved surface area and the creation of oxidative stress. The value of this research is that it is the first time to produce chitosan NCs using *Streptomyces*. In addition, the unique ability of this metallo-chitinase in chitin degradation, pH stability, and thermal resistance than many chitinolytic enzymes, it could be considered a more effective chitinolytic agent targeting phytopathogenic fungi in particular.

## Materials and methods

### Materials

Sephadex G_75_ FF (fast flow) and DEAE-Cellulose were obtained from Pharmacia Biotech (Sweden). Chitin, β-mercaptoethanol*,* SDS, EGTA, and EDTA were obtained from Sigma-Aldrich (USA). Additional reagents were of analytical degree and were purchased from regional providers.

### Collection of shrimp shell wastes and preparation of the substrate

Wastes of shrimp shells were gathered from Dammam regional fish market in Saudi Arabia. To remove the moisture, they were then sundried till constant dry weight was obtained. They were then milled to a fine powder. Chitin was extracted from the whole shrimp powder through the demineralization step and deproteinization step^39^. Colloidal chitin was then prepared by the addition of 5 g from the previous material to 88 ml of concentrated HCl and allowed to stir on a magnetic stirrer for 3 h. Exactly one liter of chilled distilled water was added with stirring for another 3 h. The suspension was left in the refrigerator at 4 °C for 24 h. The dense white pellet was collected by Buchner filtration. The paste is the colloidal chitin which was suspended in distilled water and filtration is repeated to get rid of HCl and the unwanted soluble residues. The last paste is kept at 4 °C for the next uses.

### Isolation, and maintenance of chitinolytic microorganisms

Several soil samples were obtained from different localities of Saudi Arabia. Initially, 1000 µl from appropriate dilutions were mixed with chitin agar plates consisting of colloidal chitin (10.0 g/l), NH_4_Cl (5 g/l), KH_2_PO_4_ (2.4 g/l), MgSO_4_.7H_2_O (0.5 g/l), K_2_HPO_4_ (0.6 g/l), and bacteriological agar (15 g/l). The medium was corrected to pH 7.0 and incubation was at 35 °C for up to 6 days. Cleared rings around colonies were taken as the first indication of chitin hydrolysis. Chitinolytic isolates were purified by quadrate streaking and single-cell colonies were preserved at − 80 °C into 20% (v/v) of sterilized glycerol.

### Assessment of chitinase productivity

The initial fermentation medium used for evaluation of chitinase productivity among the recovered isolates from soil samples was consisting of colloidal chitin (10.0 g/l), (NH_4_)_2_SO_4_ (5.0 g/l), MgSO_4_.7H_2_O (0.5 g/l), KH_2_PO_4_ (2.4 g/l), K_2_HPO_4_ (0.6 g/l), NaCl (0.6 g/l), FeSO_4_.7H_2_O (10.0 mg/l), ZnSO4. 7H_2_O (1.0 mg/l), and MnSO4. 7H_2_O (1 mg/l). The basal medium was corrected to pH 6.0 and then autoclaved for 15 min at 121 °C. After being cooled to below 45 °C, each 250 ml Erlenmeyer flask contained 50 ml liquid and was inoculated with 2 ml (10^7^ spores/ml) of a 7-day-old culture. Incubation was done at 35 °C for 6 days on a shaking incubator at 150 rpm speed. The microbial growth was withdrawn by filtration and then centrifugation at 5000 rpm for 20 min. Enzyme production among isolates was evaluated and the most potent isolate was selected. The nutritional and environmental parameters were then optimized to achieve the highest level of chitinase productivity. The final fermentation broth was used for enzyme purification.

### Molecular characterization of chitinolytic microorganisms

The QIAamp DNA Mini Kit (Qiagen, Germany) was employed for the extraction of bacterial DNA following the provider's guidelines. The gene coding 16S rRNA was amplified partially by employing 10 µM of each universal primer: 27F (5′-AGAGTTTGATCATGGCTCAG-3′) as forward primer and 800R (5′TACCAGGGTATCTAATCC3′) as reverse primer. The PCR was then initiated at 95 °C for 10 min, followed by 35 denaturation cycle at the same temperature for 1 min, the annealing step at 56 °C for 1.15 min, and the extension step for 2 min at 72 °C, followed by final extension step at 72 °C for 5 min. The reaction product was eluted into agarose gel electrophoresis of 1.5% agarose. The gel was dyed with ethidium bromide solution then, gel bands were visualized under UV. The PCR amplicons were purified by ExoSAP-IT (Applied Biosystems, USA) and the purified amplicons were prepared for sequencing by Big Dye® Terminator v3.1 Cycle Sequencing Kit (Applied Biosystems, USA) and DyeEx purification kit (Qiagen, Germany). The sequencing was conducted on a SeqStudio Genetic Analyzer (Applied Biosystems, USA). Consequently, the acquired 16S rRNA partial gene sequences were deposited in the GenBank database (www.ncbi. nlm.nih.gov/genbank/) after identifying species with highest similarity using National Center for Biotechnology Information (NCBI) through BLAST searches (http://blast.ncbi.nlm.nih.gov/Blast.cgi).

### Assay of chitinolytic activity and protein determination

The enzyme–substrate reaction was initiated by mixing 1 ml of 10% (w/v) colloidal chitin suspension in 0.2 M sodium phosphate buffer (pH 7.0) to 1 ml of enzyme preparation. The reaction proceeded at 50 °C then was terminated after 1 h by 1 ml of 1% NaOH and incubation in a boiling water bath (100 °C) for 5 min. Centrifugation was done at 5000 rpm for 15 min to split the product from the non-reacting substrate. Then the total reducing sugars were measured by dinitrosalicylic acid (DNS) assay^40^. Exactly, 1 ml of the supernatant was combined with 1 ml of 1% DNS in 30% sodium potassium tartrate in 2 M NaOH. The mixture was vortexed and reincubated in a boiling water bath for 10 min. The shift of color towards red is a direct indication of the presence of reducing sugars and therefore proportional to the enzymatic activity. The absorption of the test sample was measured at *A*_535_ (UV5 Excellence UV/VIS Spectrophotometers, Mettler Toledo, Switzerland). One unit (**U**) of chitinase was calculated as the quantity of enzyme necessary to yield 1 µmol of NAG as reducing sugar per minute at 50 °C. Protein quantification was done at *A*_280_ using bovine serum albumin (BSA) as a standard protein.

In some experiments, chitinolytic activity was visualized by chitin agar well diffusion plates consisting of colloidal chitin (10 g/l) and agar (15 g/l) at pH 7.0. Exactly, 10 mm diameter wells were bunched off using a sterile cork porer. Fifty-μl enzyme preparation was added to each well. Blank was done by 50 μl of a thermally inactivated chitinase preparation. Cleared halos formation was checked after 24 h at 35 °C by the addition of a thin layer of Lugol’s iodine solution on the surface of the petridish for 10 min. Simple sugars resulting from chitin degradation don’t absorb the iodine solution while undegraded chitin absorbs the dye and appears red.

### Enzyme purification

The procedure of chitinase separation from the culture broth of the most potent isolate was initiated by centrifugation at 5000 rpm for 15 min to separate the cells and debris from the bacterial products. The proteins and enzymes present in the supernatant were then precipitated by the addition of ammonium sulfate gradually with frequent stirring till 80% saturation level was achieved. After 12 h incubation at 4 °C, the agglomerated enzyme molecules were harvested by centrifugation at 7000 g for 10 min at 4 °C. The harvest was then suspended in 100 mM Tris–HCl buffer of pH 7.4 (buffer A) for dialysis against buffer A to get rid of ammonium sulfate and other salts. The resulting dialysate was then applied on the top of DEAE-Cellulose anion-exchanger column chromatography of 2.0 × 30 cm^2^ dimension. Elution was done with 100 mM borate buffer pH 9.4 (buffer B) with 0–1 M NaCl in a linear gradient manner. The active fractions from the anion-exchanger column were assembled and lyophilized for further purification with the gel permeation chromatography technique. For this, a Sephadex G_75_ FF column of dimension 2.0 × 45 cm^2^ was used. The elution through this column was done with buffer A at a 0.5 ml per minute flow rate. Again, the fractions representing chitinase activity were combined and lyophilized for characterization and application. Ultimately, SDS-PAGE was accomplished using 5% (w/v) stacking gel and 12% (w/v) separating gel to check the purity of chitinase and to verify the exact molecular mass of the tested chitinase. For zymogram analysis, the crude chitinase was mixed with the loading buffer without reducing agent and boiling step. After electrophoresis, in gel containing 1.0% colloidal chitin as substrate, the gel was immersed in 0.2 M Tris–HCl buffer, pH 8.0 at 40 °C for 16 h. Chitinase activity in the native gel was visualized by staining with Lugol’s iodine solution for 15 min and destaining with 1 N NaCl solution for 5 min at room temperature.

### Biochemical properties

#### Impact of pH on chitinase activity and stability

Chitinase-substrate reaction mixtures were corrected to various pHs. The pH range of 2.0–6.0 was achieved by the citrate–phosphate buffer, the pH range of 7.0–8.0 was achieved by Tris–HCl buffer, and pHs 9.0–11.0 were achieved by the glycine–NaOH buffer. The assay of chitinase activity was done at 35 °C using the substrate colloidal chitin. In addition, the pH stability was assessed by preincubation of chitinase alone for 60 min at 35 °C at the same pH range, then the remaining activity against colloidal chitin was assessed at pH 8.0 with 50 mM Tris–HCl buffer.

#### Impact of temperature on chitinase activity and stability

The impact of temperature on the enzyme was evaluated at 20 °C to 50 °C. The substrate colloidal chitin was used at a concentration of 1.5% (w/v). The colloidal chitin was adjusted to pH 8.0 in 50 mM Tris–HCl buffer. The heat stability of chitinase was assessed by preincubation of chitinase preparations without substrate in the same buffer at high temperatures of 50–80 °C for 15–60 min. At time intervals, the remaining activity was assessed. The temperature at which chitinase lost half of its original activity after 60 min exposure was defined as the midpoint temperature (*T*_m_).

#### Impact of metal ions, activators, and inhibitors

The purified chitinase was preincubated at 35 °C for 60 min in the existence of a metal such as Mg^2+^, Cu^2+^, Ca^2+^, Mn^2+^, Hg^2+^, Ag^+^, Fe^2+^, and Fe^3+^ in addition to common reagents such as β-mercaptoethanol*,* SDS, EGTA*,* and EDTA. They were tested at a strength of 5 mM in 50 mM phosphate buffer (pH 8.0). The remaining activity was then assessed. The activity of chitinase in the absence of metals, activators, or inhibitors was considered 100%.

### Antifungal activity

The antifungal potential of the three purified chitinases Am1, Bat2, and Nof21 was studied by the method of agar well diffusion according to the National Committee for Clinical Laboratory Standards (NCCLS)^41^. Five days-old spore suspensions (3 × 10^7^/ml) at the concentration of 1 ml of *Eurotium amstelodami* (*Aspergillus amstelodami*), *Fusarium oxysporum,* and *Fusarium verticillioides* were uniformly and spread individually on plates of Potato Dextrose Agar with pH adjusted at 5.5. The cultures were left at room temperature for 10 min to allow absorption of the inoculum. Eight-mm wells were punched off using a sterile cork borer and forty-µl of chitinase preparation was applied to each well (15 U). A heat-inactive enzyme preparation was utilized as a negative test. The growth of fungi was observed periodically at 30 °C for 7 days. Tests were conducted in three replicates to assure reliability.

#### Statistical analysis

Tests were achieved in triplicates and results were given as means ± standard deviations.

## Supplementary Information

Supplementary Information.

## Data Availability

All data generated and analyzed during this study are included in this published article. The datasets generated during the current study are available in GenBank database under accession number OP647097 for *Streptomyces mutabilis* Nof21, accession number OP647095 for *Streptomyces variabilis* Am1, and accession number OP647096 for *Streptomyces roietensis* Bat2.

## Abbreviations

NCCLS: National Committee for Clinical Laboratory Standards
DEAE-Cellulose: Diethylaminoethyl cellulose
DNS: Dinitrosalicylic acid
EDTA: Ethylenediamine tetraacetic acid
EGTA: Ethylene glycol-bis(β-aminoethyl ether)-N,N,N′,N′-tetraacetic acid
NAG: N-acetyl-D-glucoseamine
PDA: Potato dextrose agar
SDS: Sodium dodecyl sulfate
SDS-PAGE: Sodium dodecyl-sulfate polyacrylamide gel electrophoresis
*T*_*m*_ value: Midpoint temperature value
