# Supplementary material for: Screening for chitin degrading bacteria in the environment of Saudi Arabia and characterization of the most potent chitinase from Streptomyces variabilis Am1
Source: Sci Rep. 2023 Jul 20;13:11723. doi: 10.1038/s41598-023-38876-2 (PMC10359409; doi:10.1038/s41598-023-38876-2)
Supplement: Supplementary file 1 — Supplementary Information. [file 41598_2023_38876_MOESM1_ESM.docx]

**Supporting information**

**
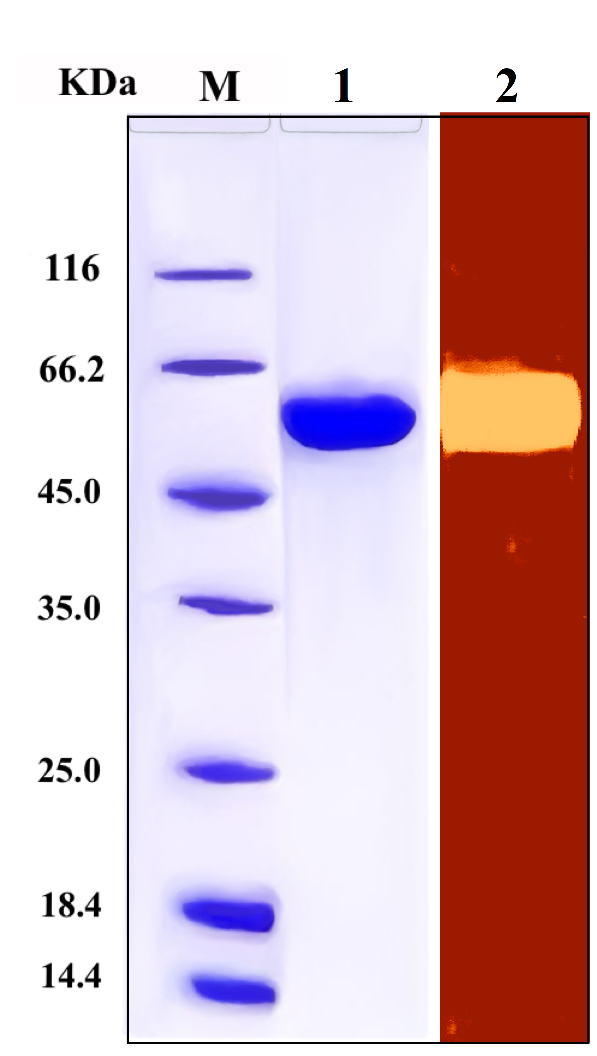
**

**S1 Fig.** The previous version of SDS-PAGE of the purified enzyme using 5% stacking gel and 12% separating gel and the zymogram of crude enzyme. M represents the standard proteins, lane 1 represents the purified enzyme on SDS-PAGE gel, while lane 2, represents the activity gel electrophoresis (zymogram). The native gel containing contained 1.0% colloidal chitin as substrate, the gel was immersed in 0.2 M Tris-HCl buffer, pH 8.0 at 40ºC for 16 hr. Chitinase activity in the native gel was visualized by staining with Lugol’s iodine solution for 15 min and destained with 1 N NaCl solution for 5 min at room temperature.
